# Effects of Nutrition on Maternal Health, Fetal Development, and Perinatal Outcomes

**DOI:** 10.3390/nu16030375

**Published:** 2024-01-27

**Authors:** Aikaterini Apostolopoulou, Antigoni Tranidou, Ioannis Tsakiridis, Emmanuella Magriplis, Themistoklis Dagklis, Michail Chourdakis

**Affiliations:** 1 Laboratory of Hygiene, Social & Preventive Medicine and Medical Statistics, School of Medicine, Faculty of Health Sciences, Aristotle University of Thessaloniki, 54124 Thessaloniki, Greece; katapost@yahoo.gr (A.A.); antigoni.tranidou@gmail.com (A.T.); mhourd@gapps.auth.gr (M.C.); 2 3rd Department of Obstetrics and Gynecology, School of Medicine, Faculty of Health Sciences, Aristotle University of Thessaloniki, 54642 Thessaloniki, Greece; dagklis@auth.gr; 3 Laboratory of Dietetics & Quality of Life, Department of Food Science and Human Nutrition, Agricultural University of Athens, Iera Odos 75, 11855 Athens, Greece; emagriplis@eatsmart.gr

## 1. Introduction

The early life theory states that the first 1000 days of a person’s life are highly influential, as lasting health impacts can be attained during this period [1,2,3]. Growing evidence has shown that optimizing maternal nutrition prior to pregnancy, including micronutrient adequacy both in the preconception period and during pregnancy is crucial for later-life health [3]. This Special Issue, “Effect of Nutrition on Maternal Health, Fetal Development, and Perinatal Outcomes”, brings together pivotal studies that address these points, shedding light on the multifaceted nature of nutrition and its implications for perinatal outcomes.

Despite certain controversies, there is an overall agreement regarding nutritional requirements during pregnancy and a focus on a balanced diet, in line with guidelines on healthy eating, to ensure the adequate intake of energy and macro- and micro-nutrients during this period [3,4]. Moreover, guidance on appropriate weight gain during pregnancy is included in the recommendations; it is generally agreed that weight gain during pregnancy should be monitored as it affects maternal and child health during and after gestation [5]. High-fat and carbohydrate diets are related to inflammatory processes that may be harmful to children’s brain development, increasing the risk of impaired cognitive function and neuropsychiatric disorders, such as depression, attention deficit hyperactivity disorder (ADHD), autism spectrum disorder (ASD), and anxiety [6,7]. In addition, the overconsumption of nutrient-poor foods may lead to another form of malnutrition due to micronutrient deficiencies [8,9] despite energy overconsumption. This condition, known as hidden hunger, is common throughout the world, mostly in high-income countries [10]. Recent evidence shows that young pregnant women are more vulnerable to this condition and could develop iron, iodine, and vitamin D deficiencies, which, in turn, are related to several pregnancy complications [10]. In adjunction to this form of malnutrition, ultra-processed food consumption has also increased in recent years and has been related to oxidative stress [1], and this may further affect pregnancy outcomes.

According to a recent meta-analysis, higher adherence to a healthy diet, characterized by high intakes of fruits, vegetables, whole grains, low-fat dairy products, vegetable oils, and fish reduced the risk of gestational hypertensive disorders, maternal depression, low birthweight, and preterm delivery [11]. On the contrary, higher maternal adherence to an unhealthy diet, characterized by refined grains, foods high in saturated fats, red meat, processed meat, fast foods, and high sugary foods or a mixed diet (combination of both healthy and unhealthy foods) was associated with a higher risk of gestational hypertension [12]. Higher intake of sugar-sweetened beverages and lower intake of oily fish were most prominently associated with higher glycemic results on the test of glucose tolerance among high-risk women [13].

Several systematic reviews have pooled together interventional studies to increase the understanding of a diet’s role in pregnancy [11,12] and indicated the importance of women’s nutrition before and during the first trimester of pregnancy. Another systematic review assessed the effects of healthy diet and exercise interventions during pregnancy and revealed a possible lower risk of gestational diabetes and cesarean section, while no clear differences were identified between groups for hypertensive disorders, perinatal mortality, or large-for-gestational-age neonates [13]. Regarding overweight or obese pregnant women, diet and/or exercise (regular aerobic exercise) interventions initiated at <21 weeks of pregnancy may reduce gestational weight gain but have no effect on the risk of hypertensive disorders of pregnancy [14]; diet interventions included dietary advice or related interventions in pregnancy that aimed to optimize health outcomes, which might include controlling excessive gestational weight gain or improving glycemic control.

## 2. An Overview of Published Articles

A Greek study developed a model to be used as an early prediction tool for gestational diabetes mellitus (GDM) risk, combining maternal characteristics, obstetric and medical history, and early pregnancy-specific biomarker concentrations, readily available in healthcare settings (Contribution 1). The model can be used for the proactive management of GDM, allowing for timely nutritional and lifestyle interventions that can significantly alter the course of pregnancy and fetal development, as well as the perinatal outcomes.

In addressing the broader spectrum of maternal health, a cross-sectional study in Poland emphasized the importance of physical activity both before and during pregnancy, highlighting its positive impact on reducing adverse perinatal outcomes (Contribution 2). The key discoveries from this research indicate that engaging in physical activity both before and during pregnancy, along with maintaining a normal pre-pregnancy body mass index (BMI), can have a positive impact on pregnancy outcomes. This influence is manifested in a decreased risk of preterm birth rates and a lower likelihood of delivering children with low birth weight. 

Another critical area of concern, as highlighted in a study focusing on Romanian women of reproductive age, is obesity (Contribution 3). The study provides a comprehensive analysis of BMI trends over 12 years, revealing a worrying increase in overweight and obesity rates. The findings indicate that, during the first-trimester morphology scan evaluation, 29% of the participants were either overweight or obese, while the rates of overweight and obese women exceeded 40% in the second trimester of pregnancy. In this study’s population, the association of obesity with other risk factors showed an elevated risk of obesity among multiparous individuals, those who smoked or were ex-smokers in the second trimester, and participants who did not take folic acid or multivitamin supplementation.

Furthermore, in Indonesia, a mentoring program implemented throughout pregnancy as an intervention yielded superior outcomes in terms of fetal growth and neonatal birth weight (Contribution 4). Notably, women who participated in the program exhibited a significantly higher weight-for-length Z-score (WLZ) for their newborns, although there was no significant difference in the length-for-age Z-score (LAZ) compared to those who received standard care alone. This innovative approach, focusing on preconception and pregnancy care, demonstrates significant improvements in fetal growth and neonatal birth weight, offering a promising strategy for enhancing maternal and child health in low- and middle-income countries. 

## 3. Conclusions

The ongoing pandemic of “hidden hunger”, characterized by excessive consumption of ultra-processed and nutrient-poor foods, is prevalent in Western countries, particularly among young individuals. While evidence on maternal nutrition predominantly focuses on low-income countries, emphasizing the detrimental effects of malnutrition on fetal growth, research examining the impact of “hidden hunger” on fetal development remains insufficient and lacks robustness to significantly influence medical decisions and interventions for young pregnant women. This Special Issue has the potential to significantly contribute to filling these existing gaps.

In conclusion, this Special Issue captures the diversity and complexity of research on nutritional impacts on maternal health, fetal development, and perinatal outcomes. The range of methodologies and subjects covered in these articles reflects the dynamic nature of this field. From predictive modeling to practical interventions, these studies significantly contribute to our understanding and management of maternal and fetal health. This compilation not only provides valuable insights into current research but also sets the stage for future investigations in this vital area of public health. More research is needed to explore personalized nutritional strategies grounded in contemporary scientific knowledge for enhancing maternal health and optimizing perinatal outcomes.
